# Healing of Early Stage Fatigue Damage in Ionomer/Fe_3_O_4_ Nanoparticle Composites

**DOI:** 10.3390/polym8120436

**Published:** 2016-12-15

**Authors:** Wouter Post, Ranjita K. Bose, Santiago J. García, Sybrand van der Zwaag

**Affiliations:** Novel Aerospace Materials, Faculty of Aerospace Engineering, Delft University of Technology, Kluyverweg 1, 2629 HS Delft, The Netherlands; w.post@tudelft.nl (W.P.); r.k.bose@tudelft.nl (R.K.B.); s.vanderzwaag@tudelft.nl (S.v.d.Z.)

**Keywords:** self-healing, poly(ethylene-*co*-methacrylic acid) ionomers, fatigue damage, inductive heating, polymer nanoparticle composites

## Abstract

This work reports on the healing of early stage fatigue damage in ionomer/nano-particulate composites. A series of poly(ethylene-*co*-methacrylic acid) zinc ionomer/Fe_3_O_4_ nanoparticle composites with varying amounts of ionic clusters were developed and subjected to different levels of fatigue loading. The initiated damage was healed upon localized inductive heating of the embedded nanoparticles by exposure of the particulate composite to an alternating magnetic field. It is here demonstrated that healing of this early stage damage in ionomer particulate composites occurs in two different steps. First, the deformation is restored by the free-shrinkage of the polymer at temperatures below the melt temperature. At these temperatures, the polymer network is recovered thereby resetting the fatigue induced strain hardening. Then, at temperatures above the melting point of the polymer phase, fatigue-induced microcracks are sealed, hereby preventing crack propagation upon further loading. It is shown that the thermally induced free-shrinkage of these polymers does not depend on the presence of ionic clusters, but that the ability to heal cracks by localized melting while maintaining sufficient mechanical integrity is reserved for ionomers that contain a sufficient amount of ionic clusters guaranteeing an acceptable level of mechanical stability during healing.

## 1. Introduction

Polymer based composites are susceptible to many different types of mechanical damages which reduce their reliability and potentially decreases the overall lifetime of the material. By implementation of self-healing technologies the overall lifetime of polymer composites can be prolonged [1,2]. Within self-healing composites, most attention so far has been on extrinsic healing strategies where an external (liquid) healing agent capable of restoring either the matrix or the filler-matrix interface is encapsulated and embedded in the matrix [3,4,5]. The mechanism certainly works but there are many issues still to be resolved. Even when solved, the fact remains that the healing reaction locally works only once and this is a major shortcoming [2]. The use of intrinsically healing polymer matrices in such composites is considered to be more optimal because it has the potential of an infinite amount of healing cycles. Additionally, intrinsic healing leaves the optimized macroscopic fiber and ply architecture required for high level mechanical properties unaffected [2,6,7].

Ionomers are among the most frequently studied polymer matrices for intrinsic self-healing particulate [8,9] or fiber reinforced composites [10]. Ionomers have pendant acid groups distributed along the polymer backbone that are neutralized by ionic metal salts. These ionic groups have the tendency to form ionic clusters which create additional physical crosslinks within the polymer network [7]. Ionomers have proven to be capable of restoring mechanical stability by healing of ballistic impact damage using a combination of shape recovery (sometimes called “shape memory”) and re-bonding across former damage site surfaces [11,12,13,14,15]. The combination of the shape recovery effect and healing is not exclusive for ionomers but is also found in other polymer systems [16,17]. Besides healing after ballistic impact, which is investigated in the majority of self-healing ionomer studies, ionomers were also used to heal scratch damage [18] and damage on composite toughening interlayers [19]. The shape restoration of the polymer after puncture is made possible by the heat that is generated upon impact [13,14]. Strain recovery after deforming a polymer beyond its yield strain and subsequent heating is found to be typical for all semi-crystalline polyethylene-based polymers [20,21] and is attributed to the dominance of the decrease in the carbon bond angle over the overall carbon-carbon stretching when these polymers are deformed and heated consecutively [22,23]. Semi-crystalline ionomers were also reported to behave like traditional shape memory polymers by Dolog and Weiss [20]. Since this form of thermal contraction after deformation does not correspond to the definition of shape memory polymers, the phenomenon was more accurately defined as free-shrinkage [21] and we will use this terminology in the present work. Although there seems to be consensus about the mechanisms responsible for the restoration after polymer deformation, there is currently no general agreement on the role that the ionic clusters have on the healing effect [24], i.e., the reformation of mechanical strength across a former crack.

As is the case for the majority of the intrinsically healing polymers, ionomers need a thermal stimulus to activate their healing behavior. This poses a direct disadvantage in future applications when the intended energy input has to be delivered from the nearby environment to the composite structure (e.g., by using an oven) [25,26,27]. To overcome this disadvantage the energy input can be delivered locally from within the structure by making the ionomer suitable for inductive heating. In recent years this concept was explored by adding ferromagnetic particles to thermoplastic matrices [28,29,30]. However, within these studies the thermoplastic material was simply melted and restored to its initial shape and lost all mechanical stability throughout the process. Recently, Hohlbein et al. demonstrated the concept of inductive healing in a new family of ionomers [8]. Although this study showed the great potential of inductive heating for intrinsically self-healing polymers, their experimental ionomers still had rather low tensile properties.

In most studies on self-healing polymer composites, the research focused on the healing of damage after cutting or static overloading [2,7]. However, when a self-healing polymer is incorporated into a structural composite it is crucial to understand how the material behaves under dynamic fatigue loading and what types of damage are formed during the early stages of this process when the likelihood of complete healing is highest. Multiple studies describe the self-healing of fatigue induced mechanical damage in extrinsic healing composites [31,32,33,34,35]. A recent study focused on the partial restoration of the functional piezoelectric properties in a lead zirconium titanite (PZT) ionomer composite [9]. Nevertheless, to the best of our knowledge, the restoration of mechanical properties after fatigue in intrinsically self-healing polymers has not been investigated.

This study is the first investigation on the self-repair of mechanical properties of intrinsically self-healing polymer particulate composites after fatigue loading conditions. In this work, poly(ethylene-*co*-methacrylic acid) zinc ionomer/Fe_3_O_4_ nanoparticle composites were developed and subjected to different levels of fatigue loading. The initiated early stage fatigue damage was then healed upon localized heating of the particles by exposure of the composites to an alternating magnetic field. For a proper understanding of the mechanisms involved in the healing process a detailed thermo-mechanical investigation was performed on a set of poly(ethylene-*co*-methacrylic acid) based polymer blends with varying amounts of ionic clusters. Such an approach allowed the identification and separation of the two stages involved in the healing process: (i) the residual strain and network restoration; and (ii) the macroscopic crack sealing. A temperature window for the different stages of early stage damage healing in ionomer composites was thereby identified.

## 2. Materials and Methods

### 2.1. Materials

In order to evaluate the effect of cluster content, four different poly(ethylene-*co*-methacrylic acid) (EMAA) zinc ionomer blends were prepared based on a previous study that investigated the role of free carboxylic content and cluster state on the healing of surface scratches [18]. The four chosen blends resulted in polymer systems with high (Zn-EMAA), medium (Zn-EMAA/EMAA), no ionic groups (EMAA) and a blend where a relatively high amount of ionic clusters is neutralized (ZnEMAA/AA). In order to make the blends susceptible to inductive heating Fe_3_O_4_ particles (10 vol %, 50–100 nm, Sigma Aldrich, Zwijndrecht, The Netherlands) were added to the polymers based on previous healing studies. More information about the nature of the polymers and particles and full characterization can be found elsewhere [8,14,20,21,29]. The selected blends were prepared with the following materials:

- Zn-EMAA: Surlyn 9520^®^(Dupont™) containing 3.5 mol % methacrylic acid groups (MAA) out of which 71% were neutralized with Zn^2+^ ions.
- EMAA: Nucrel 960^®^ (Dupont™) containing 5.4 mol % of MAA groups.
- Zn-EMAA/EMAA: 50/50 wt % blends of Zn-EMAA and EMAA.
- Zn-EMAA/AA: 90/10 wt % blend of Zn-EMAA with adipic acid (AA) powder (Sigma Aldrich). In this blend the ionic clusters are destroyed by the adipic acid as is described by Varley et al. [36].

Polymer composites were prepared by mixing all components (polymer pellets, particles and additives) using a twin screw mini-extruder. The extruder volume was 15 mL and a temperature of 200 °C and a torque of 50 rpm were applied. The residence time in the extruder was 5 min. After extrusion, the resulting products were compression moulded at 150 °C with a pressure of 4 MPa using a hot press resulting in 100 ± 5 µm freestanding films. Teflon films were used to separate the polymer films from the pressing plates. After moulding, the films were given a 15 min heat treatment at 80 °C in a preheated convection oven to equilibrate the thermal effects induced due to the rapid cooling after moulding. Films were stored at room temperature for at least 21 days to equilibrate the polymer microstructure prior to further testing. Dog-bone shaped specimens (ASTM D1708) were pressed from the prepared films.

### 2.2. Mechanical Testing

To study the deformation before and after free shrinkage, different levels of quasi-static strain (25%–100%) were applied to deform the polymer composites using an Instron Model 3365 universal testing systems equipped with a 1 kN load cell. Dog-bone micro-tensile specimens were stretched at 1 mm/s at room temperature. The average value of 3 experiments was reported.

Fatigue experiments were conducted on dog-bone shaped specimens at room temperature on an MTS 831 Elastomer test system equipped with a 1 kN load cell. The specimens were fatigue tested under different prestrain levels of 25% and 50% from which a sinusoidal waveform with an amplitude of 2.3% and a frequency of 1 Hz was employed. The amount of applied strain cycles ranged from 500 to 50,000. Full fracture tensile tests were performed on different Zn-EMAA specimens at different stages of the fatigue restoration process using the same equipment and conditions as for the deformation experiments. True stress and true strain were calculated from these tests via:

(1) $$\sigma_{T}=\frac{P}{A_{0}}\left(1+\varepsilon \right)$$

(2) $$\varepsilon_{T}=ln\left(1+\varepsilon \right)$$

where, σ_T_ = true stress in MPa; *P* = measured load in N; *A*_0_ = Area of the cross-section of the dog-bone in mm^2^; ε = engineering strain in percent; and ε_T_ = true strain in percent.

The tensile tests were performed 7 days after the fatigue and healing treatments which allows the polymer crystalline phases to fully recover prior to further testing.

### 2.3. Thermomechanical Testing

The effect of cluster content on the deformation that occurs during fatigue and the thermal contraction upon heating was investigated using a Perkin–Elmer Sapphire differential scanning calorimeter (DSC). Samples were heated and cooled between −50 °C and 150 °C at a rate of 20 °C/min under a nitrogen atmosphere.

To obtain a deeper understanding of the effect of the clusters on the self-healing mechanism, the macroscale network mobility of the non-particulate polymer blends was investigated by oscillatory shear rheology. Experiments were performed with a Haake Mars III rheometer. An 8 mm diameter (stainless steel) parallel plate geometry was used throughout. For all the samples, the polymer thickness was between 0.9–1.2 mm, and a constant shear strain *γ* of 1%, which was within the linear viscoelastic regime of the materials, was applied. Frequency sweep experiments between 10^2^ and 10^−2^ Hz were performed at temperatures of 80 and 110 °C, with an isothermal hold for 20 min prior to each temperature step. The supramolecular bond lifetime (τ_b_) at different temperatures was then calculated as inverse of the frequency at which the storage and loss moduli crossover in a frequency sweep experiment.

### 2.4. Thermally Induced Healing Process and Evaluation

**Figure 1:**
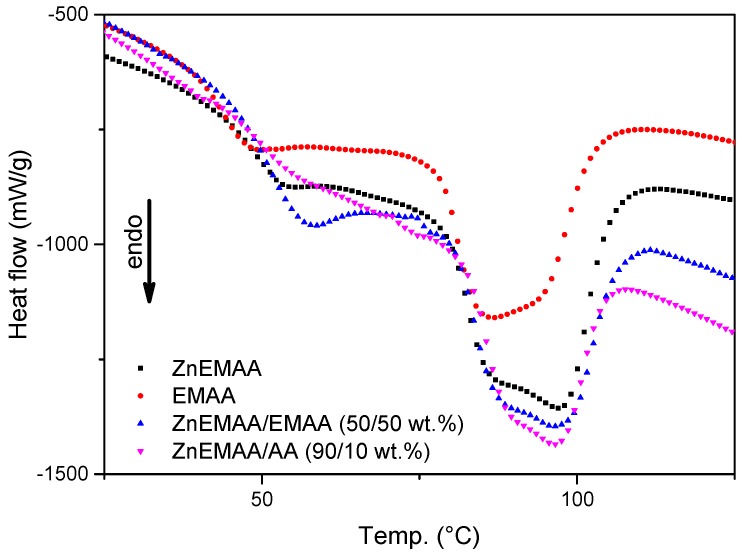
DSC thermograms for four particulate polymer blends showing the effect of ionic cluster content on the low-temperature endotherm in the melting range of the polymer composites.

**Figure 2:**
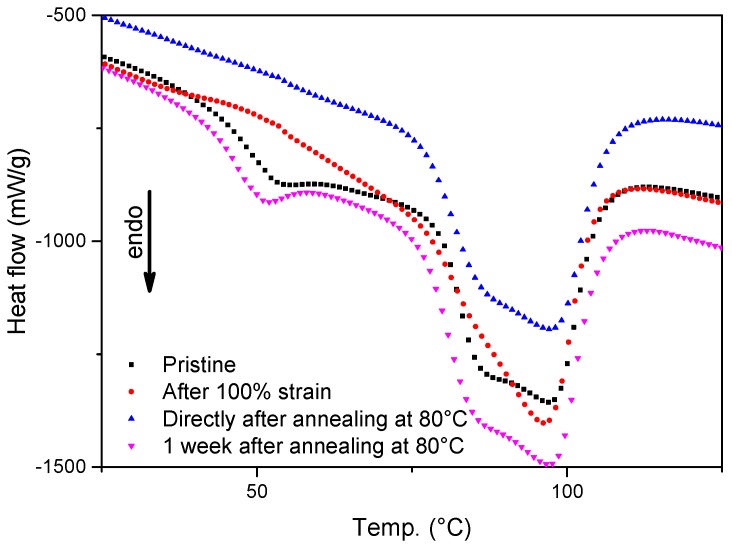
DSC thermograms of the Zn-EMAA-Fe_3_O_4_ composite at different stages of the deformation and thermal treatment process.

Induction heating was applied for 15 min using a single-turn hairpin induction coil mounted on an Ambrell Easyheat device. The coil and specimen were separated by Teflon foil and the coupling distance was fixed at 1 mm. A frequency of 350 kHz and currents between 200 and 250 A were applied to reach the intended temperatures. Healing temperatures were selected based on the different thermal transitions of the polymer as shown in Figure 1 and Figure 2. As such, the selected healing temperatures are located below the secondary thermal transition (50 °C), in between the secondary transition and the overall melting of the polymer (80 °C) and above the overall melting of the polymer (100–110 °C). The specimen temperature upon inductive heating was monitored with a FLIR A655sc infrared camera. Since this method only detects the surface temperature of the ionomer composites, a COMSOL Multiphysics model was used to derive a relation between the measured surface temperature and the desired healing temperature within the bulk of the polymer sample. The used model is a stationary heat transfer model that correlates the measured surface temperature to the bulk healing temperature based on the thermal conductivity of the materials used. The model assumes a uniform distribution of particles within in cubic geometry corresponding to the used particle concentration of 10 vol %. Full information on the applied model (geometry, input parameters and calculations) can be found in Supplementary Materials S1.

The closure and sealing of fatigue induced cracks was monitored with a digital microscope Keyence VHX2000 with a wide-range zoom lens (100×–1000× magnification). For the optimal illumination of the black surfaces the microscope was equipped with a OP-87229 short ring-light. The length of the samples before and after the thermal treatment was measured with a digital caliper and the residual strain was calculated based on this data.

## 3. Results

### 3.1. Thermal and Thermomechanical Analysis

DSC thermograms for all composite grades during heating from 25 to 125 °C are shown in Figure 1. This temperature region shows a broad melting range which includes a low temperature endotherm that typically appears between 50 and 75 °C for all four compositions. Figure 2 shows the thermograms of the Zn-EMAA/Fe_3_O_4_ composite in four different stages of the deformation and thermal treatment process: (i) material in its pristine state; (ii) after 100% strain deformation; (iii) after 100% strain and 15 min furnace annealing at 80 °C; and (iv) after 100% straining, 80 °C annealing and 1 week storage at room temperature.

Figure 1 and Figure 2 show the effect the low temperature endotherm upon ionic cluster concentration and during the process of free-shrinkage respectively. In recent literature, the endotherm has often been attributed to a declustering of the ionic clusters which would lead to enough mobility within in the polymer network to support healing [8,9,37,38]. Other studies claim that the endotherm corresponds to the glass transition temperatures of the various phases within the polymer (matrix phase T_g_ < 0 °C) that are linked to the ionic cluster concentration [39,40]. Figure 1 shows that this endotherm also exists in the non-ionic EMAA and is only intensified upon the addition of ionic groups within the polymer grade. The addition of adipic acid results in the diminishing of this endotherm as was reported previously [14]. Figure 2 shows that the endotherm disappears upon straining and returns only after one week of annealing at 80 °C and is therefore not present during the process of free-shrinkage.

**Figure 3:**
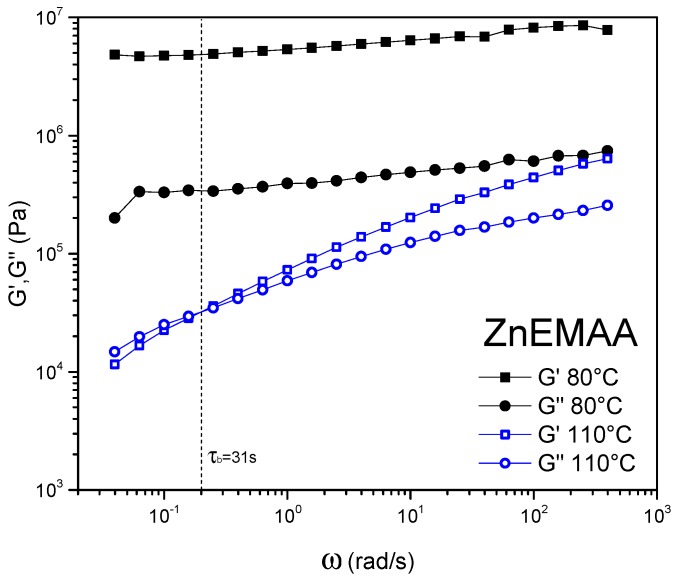
Frequency sweep experiment of the Zn-EMAA polymer at 80 and 110 °C. The supramolecular bond lifetime τ_b_ is determined for both polymers at intersection of the *G*’ and *G*” at 110 °C.

Figure 3 shows the storage (*G*’) and loss moduli (*G*’’) of Zn-EMAA in the frequency range of 10^2^–10^−2^ Hz obtained by frequency sweep rheology. Similar curves were obtained for the other polymer grades. For all polymer grades it is found that at 80 °C the storage and loss moduli curves do not intersect and therefore no values of τ_b_ can be determined. *G*’ and *G*” were found only to intersect at temperatures close to of 110 °C which is the overall melting temperature of the polymer grades. The plateau modulus (*G*_N_), which is taken as the high frequency plateau of the *G*’ curve was used to compare the mechanical robustness of each sample. In one of our recent publications we showed the connection between the macroscopic network mobility of ionomers with varying amounts of ionic clusters to the supramolecular bond lifetime (τ_b_). It was then proposed that a polymer system with 10 s < τ_b_ < 100 s and 10^5^ Pa < *G*_N_ < 10^7^ Pa would show good healing behavior combined with strong mechanical properties [41]. The values for *G*_N_ and τ_b_ of the four polymer blends are presented in Table 1. Because the final plateau modulus at 110 °C was beyond the high frequency range of the rheometer, the *G*’ values for the highest measured frequency are reported instead.

Table 1 shows that at 110 °C, the τ_b_ and *G*_N_ values of the Zn-EMAA ionomer meet the demands for good healing (10 < τ_b_ < 100 s) and good mechanical properties (*G*’ > 10^5^ Pa and is expected not to exceed 10^7^ Pa) [41]. Experiments at higher temperatures (>130 °C) move the value of τ_b_ towards the regime of viscous flow (τ_b_ < 10 s) indicating that good healing conditions are not met at temperatures well above the overall melting point of the polymer. The values found for the EMAA polymer at a temperature of 110 °C are also typical for viscous flow of a molten polymer and therefore the damage recovery cannot be classified as healing. The τ_b_ and *G*_N_ values for the Zn-EMAA/EMAA and Zn-EMAA/AA show that the thermomechanical behavior at the measured temperatures is in between that of Zn-EMAA and EMAA indicating that the difference in viscoelastic behavior is linked to the presence of ionic clusters.

### 3.2. Effect of Temperature Post-Treatment after Static and Dynamic Loading

**Figure 4:**
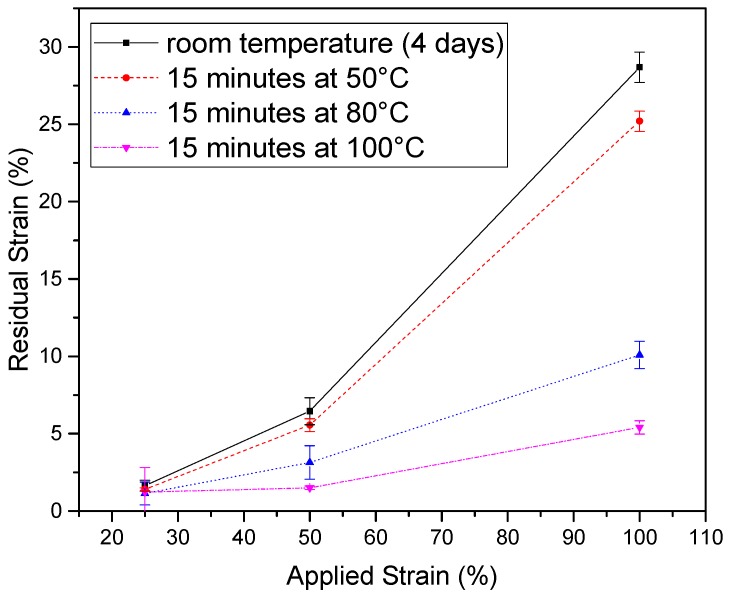
Residual contraction behavior in Zn-EMAA/Fe_3_O_4_ composites as a function of the applied static prestrain and four annealing conditions.

All prestrained polymer composites were post treated at different temperatures. As a consequence, a macroscopic shrinkage was observed and quantified. Figure 4 shows the influence of temperature on the free-shrinkage behavior of the Zn-EMAA particulate composite as function of the applied strain. Different levels of initial quasi-static strain levels were applied and the residual strain (at room temperature) after annealing at various temperatures was determined as described.

Figure 4 shows that, with certain annealing conditions, the residual strain of the Zn-EMAA polymer grade can become near zero up to an applied strain level of about 50%. This upper limit for full strain recovery turns out to be applicable for all polymer composite grades and was therefore used as the maximum prestrain level for the fatigue experiments.

**Figure 5:**
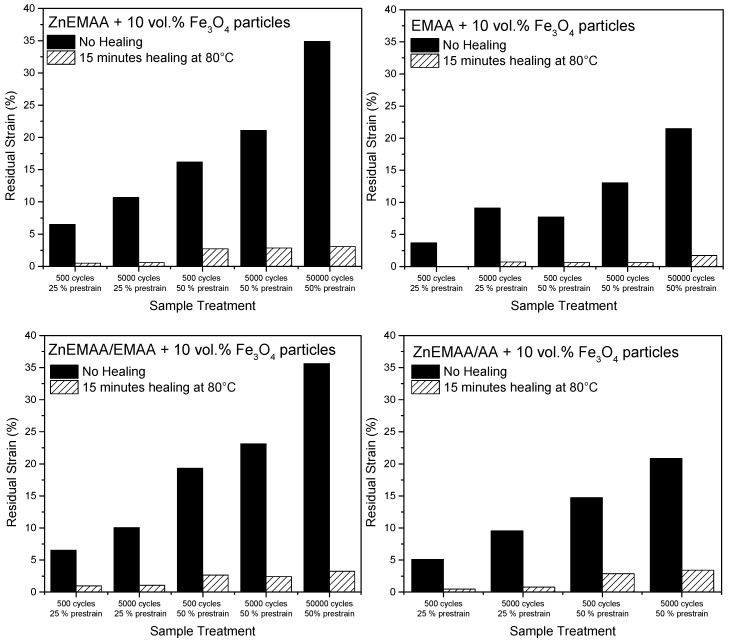
Strain restoration after fatigue treatments of Zn-EMAA, EMAA, Zn-EMAA/EMAA and Zn-EMAA/AA composites. Results are shown for different treatments based on the amount of strain cycles and the prestrain that was applied to the composites.

Figure 5 shows the residual strain of all polymer grades after different fatigue treatments before and after heating. This figure shows that the residual strain after fatigue increases when the prestrain and the number of cycles are increased. Upon a healing treatment of 15 min at 80 °C, the residual strain is reduced to levels below 5% for all investigated blends. The EMAA composite without ionic clusters has the lowest levels of residual strain before and after healing. The levels of residual strain for the Zn-EMAA, Zn-EMAA/EMAA and the Zn-EMAA composites are fairly comparable with the exception of the value for the Zn-EMAA/AA blend for which the level of contraction could not be measured after 50,000 strain cycles as complete sample failure occurred at this level of cyclic loading.

**Figure 6:**
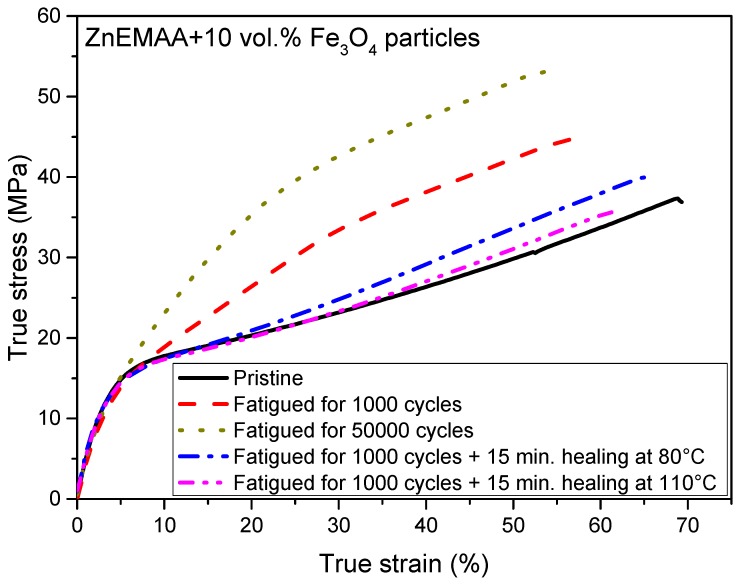
Stress–strain curves taken during the several stages of the fatigue damage-recovery process. It shows that the strain hardening increases when the number of applied fatigue cycles is increased from 1000 to 50,000 and that the original stress–strain relation can be restored when a heat treatment is applied.

Figure 6 shows the stress strain curves of a Zn-EMAA/Fe_3_O_4_ composite after several treatments: the quasi-static tensile behavior of a pristine specimen, two fatigued specimens at 1000 and 50,000 strain cycles with a strain amplitude of 2.3% on top of a prior 50% static strain and two specimens that were subjected to 1000 fatigue cycles and subsequently heated to either 80 or 110 °C. The obtained results indicate that a fatigued ionomer system shows strain hardening and becomes slightly less ductile. The first effect can be explained by an alignment of polymer chains that were originally packed in the secondary clusters. This explanation is supported by the DSC thermograms in Figure 2 that show that this phase disappears upon straining. This effect increases the tensile strength of the polymer composite and can therefore by itself not be seen as a damaging event. However, the second effect is an indication for the loss of mechanical integrity and can be a result of local mechanical damage in the form of random chain scission [42] which could potentially occur upon the application of multiple fatigue cycles. The figure shows that the strain hardening increases when the number of applied fatigue cycles is increased from 1000 to 50,000. Figure 6 also shows that the original stress–strain relation can be restored when a suitable heat treatment is applied. A heat treatment of 80 °C already shows a big reduction of the strain hardening effect and after a 110 °C treatment the initial tensile behavior is almost completely restored.

**Figure 7:**
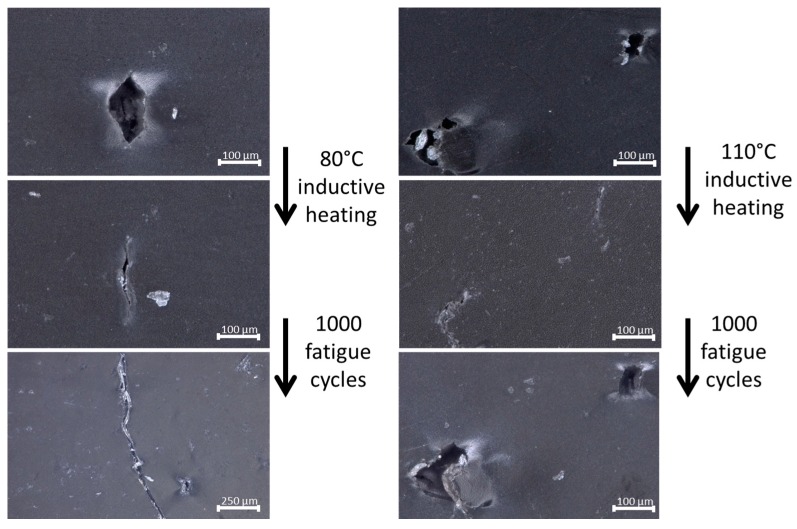
OM images showing the difference between crack closure and crack sealing which is achieved by inductive at different temperatures: (**left**) the crack closure at 80 °C and the further crack propagation upon a second treatment of 1000 fatigue cycles; and (**right**) a series of cracks that are sealed at 110 °C and reopened upon a second fatigue treatment of 1000 fatigue cycles.

Optical microscopy images of the surface of a fatigued Zn-EMAA specimen (1000 strain cycles, 50% prestrain) before and after inductive heating are shown in Figure 7. The analysis showed that some of the nanoparticles formed micron-sized agglomerates rather than being homogeneously distributed which suggests that the results currently obtained are not fully optimal. The agglomerates promote the crack initiation upon straining and fatigue loading, but their presence does not disturb the mechanism of fatigue healing to be demonstrated in this work. The images show that fatigue loading led to the formation of microcracks close to clusters of Fe_3_O_4_ particles which act as stress concentrators in the composite. A similar fatigue treatment on a Zn-EMAA specimen without particles did not show any microcracks. The images also show that inductive heating at 80 °C closes the cracks but does not seal the edges of the crack back together. Upon a second fatigue treatment of 1000 cycles, it is shown that these cracks propagate into larger cracks. On the other hand, inductive annealing at 110 °C shows a complete sealing of the crack edges and results in the effective disappearance of the crack. In this case, a follow-up fatigue treatment only leads to reopening of these cracks. This is expected since the crack locations remain the weak spots of the composite. However, after a 110 °C induction treatment the cracks have not propagated as is seen for the specimens that are only healed at 80 °C. These observations are in line with the results of the frequency sweep experiments obtained in Figure 3.

**Figure 8:**
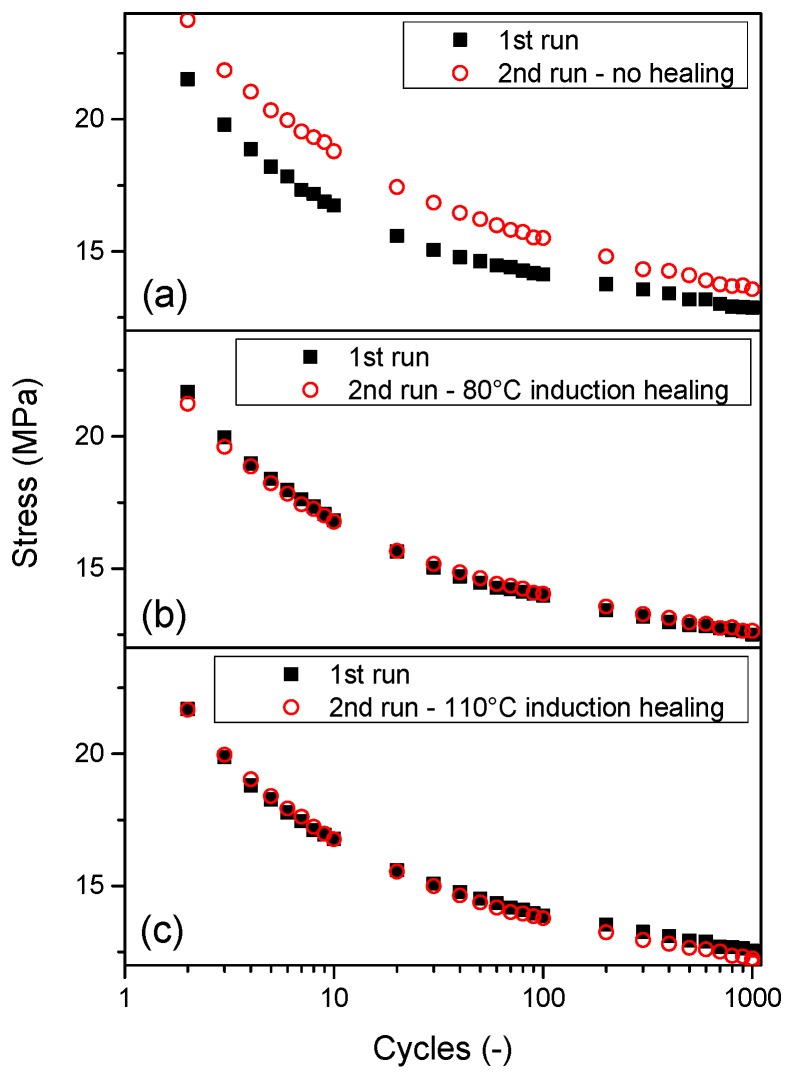
Fatigue response of three Zn-EMAA/Fe_3_O_4_ composites after two consecutive experiments. The first run in all three graphs is performed on a pristine specimen and the second run shows the response: (**a**) after one week without additional treatments; or after 15 min of inductive heating at: (**b**) 80 °C; and (**c**) 110 °C.

Figure 8 shows the decay in maximal stress during 1000 fatigue cycles for Zn-EMAA preloaded to an initial strain of 50%. Figure 8a shows an ionomer composite specimen that was tested twice without any healing treatment in between. In this figure the strain hardening effect that is also visible in Figure 6 can be observed. Figure 8b,c shows a similar set of experiments, however, with an inductive heat treatment at 80 or 110 °C, respectively, in between cyclic loading. The figures show that both heat treatments restore the initial fatigue response and delete the strain hardening effect as a result of the recovery of the original network properties.

## 4. Discussion

The optical microscopy images in Figure 7 show a clear distinction between the closure and the sealing of fatigue induced cracks at the two healing temperatures. Although there seems to be an agreement on the mechanisms that are responsible for the contraction/closure [20,21], there is still an ongoing debate on the mechanism that is responsible for the crack sealing behavior of ionomers. The main discussion revolves around the low-temperature endotherm that is visualized by DSC in Figure 2. The majority of studies on self-healing ionomers attribute this endotherm to a declustering of the ionic multiplets that are formed within the polymer microstructure as was described by Tadano et al. [38]. It is reported that the declustering of these multiplets would create sufficient mobility for the polymer to heal at temperatures below the melting point [8,9,14,18,37]. Another theory, posed by Eisenberg, describes the clusters of multiplets as a thermally stable phase with its own T_g_ that is higher than that of the surrounding non-ionic polymer phases. In their work, the origin of the low temperature endotherm is attributed to the crystallization of secondary crystals which form in between the primary crystal lattices over time [39,40]. In a recent publication by Kalista et al., it is reviewed that the most experimental evidence points towards the latter explanation for the thermomechanical behavior of ionomers. However, the precise mechanism responsible for the self-healing of ionomers is still under discussion [24].

The results that are depicted in this work support the theory of Eisenberg over that of Tadano. A first indication is the fact that the DSC spectrum in Figure 2 shows a low temperature endotherm for the EMAA polymer. Since there are no ionic clusters present in this polymer, the endotherm in this spectrum cannot be attributed to ionic multiplet formation within the structure. The presence of ionic clusters does, however, affect the formation of the low temperature endotherm and the secondary crystalline phase as is described by Loo et al. [40]. In a similar fashion, the addition of adipic acid restricts the formation of this secondary crystalline phase as the corresponding endotherm peak around 50 °C in Figure 1 flattens out completely. The results depicted in Figure 3 also contradict the declustering concept since no crossover point between *G*’ and *G*” can be found at 80 °C [8,9,37].

The fact that the low temperature endotherm disappears upon straining (Figure 2) indicates that the molecular origin of this endotherm is not the sole explanation of the ionomer healing characteristics. The free-shrinkage behavior that is observed in this temperature range is most likely a result from the overall melting peak in the polymer which is very broad and starts at the onset of the low temperature endotherm. This statement is supported by the temperature dependency of the residual strain shown in Figure 4. The smaller crystals melt at lower temperatures while the larger crystals remain crystallized and serve as a rigid internal structural entity as is also common in shape memory polymers [20].

The thermal contraction is shown to be independent of the presence of clusters and the low-temperature endotherm, since Figure 5 shows that the strain restoration after fatigue is clearly present for all compositions. As a matter of fact, these diagrams show that the contraction is highest in the non-ionic EMAA material as only in this material 100% restoration is observed after an applied strain of 50%. This is an indication that the presence of clusters might even restrict the mobility of the reforming secondary crystal phases of the polymer and thereby hindering the free-shrinkage capacity which is supported by the studies of Loo et al. [40].

The optical microscopy images show that sealing of the fatigue induced microcracks only occurs when the ionomer is heated above 110 °C. This is in line with rheological data obtained in Figure 3 and Table 1. Here it is shown that the viscous component of the polymer does not get dominant over the elastic component before the overall melting point is reached. However, both thermal treatments lead to a full restoration of the original tensile behavior and fatigue response. This indicates that the polymer network is effectively repaired at temperatures below the melt temperature and that the formation and presence of microcracks does not directly affect the mechanical properties in the early stages of the damage formation. Nevertheless, the healing of the early stage damage will be necessary to extend the lifetime of the ionomer composites since these unsealed microcracks will eventually propagate into larger cracks, as was shown in Figure 7. These propagated cracks will ultimately induce the destructive failure of the material as was observed for the 50,000-cycle fatigue treatment of the Zn-EMAA/AA blend.

The addition of the nanoparticles (10 vol %) was found to barely affect the overall tensile properties of the polymer. Full information on the impact of the nanoparticles on the tensile properties can be found in Supplementary Materials S2. Besides a slight increase in yield strength and Young’s modulus, the main effect was an increased brittleness which is considered to have no effect on the applicability of the polymers since a tensile strain of at least 100% can still be achieved for the composites, as is depicted in Figure 4. Nevertheless, it was found that the Fe_3_O_4_ particles induce microcracks that are not observed in the pure polymer films. Based on this, it could be reasoned that the particles only weaken the material and no additional mechanical benefits are obtained. However, since the microcracks do not affect the overall tensile properties and fatigue response of the polymer composite and can be fully healed by heating at 110 °C there is a zero net negative effect of the particles on the polymer behavior. On the other hand, the ferromagnetic particles allow the polymer to be healed by inductive heating which is crucial for larger composite structures that cannot be heated by external contact heating and therefore require internal heating.

**Figure 9:**
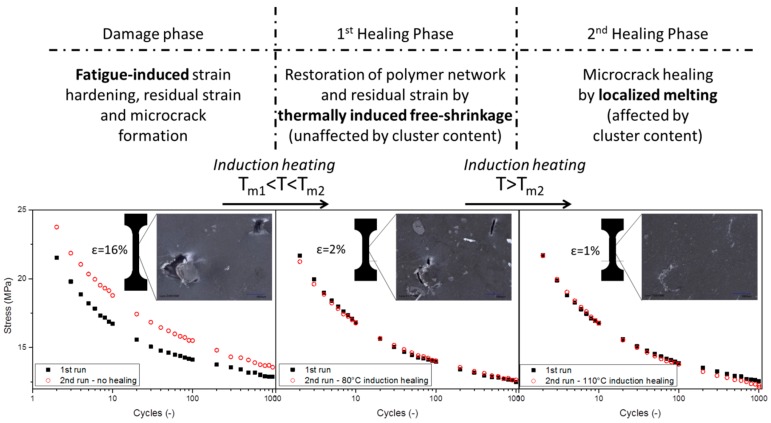
Ionomer healing temperature dependency scheme. In the 1st healing phase, the thermally induced free-shrinkage restores the residual strain and the polymer network at temperatures in between the melting point of the secondary crystal phase (T_m1_) and the overall melting point (T_m2_). In the 2nd healing phase, microcracks are closed due to localized melting above T_m_ in which the ionic clusters act as a stable phase providing sufficient mechanical properties for good healing conditions.

Although the thermal behavior below the overall melting temperature is comparable for all investigated blends and therefore independent of cluster content, there is a clear difference in the region above the melt. Table 1 shows different values for τ_b_ and *G*_N_ for the four polymer systems which can be explained by the presence of the ionic clusters. These create an additional phase in the polymer microstructure which has higher thermomechanical stability than the surrounding polymer phase. As a result, the non-ionic phase can flow in between the ionic clusters and thereby heal cracks and interfaces at a temperature above its melting point, while the overall polymer system maintains its required level of mechanical stability. When the cluster concentration is not high enough, the polymer will show melt flow and is therefore not considered to be a self-healing polymer. Based on the current observations it is possible to propose an ionomer healing temperature dependency scheme. Figure 9 shows a two-step healing mechanism in which the thermally induced free-shrinkage is independent of cluster content and can be triggered by applying a temperature between the two main melting points of the polymer. At this temperature, the residual strain and the strain hardening that occur upon deformation are fully restored. Early stage damage in the form of fatigue induced microcracks can be subsequently healed by melting the polymer while the ionic clusters act as a stable phase providing sufficient mechanical properties for good healing conditions.

## 5. Conclusions

This work reports on the healing of early stage fatigue damage in poly(ethylene-*co*-methacrylic acid) based nanoparticulate composites upon localized inductive heating. It is found that there are three main damage modes that occur in the early stage of the fatigue process: residual strain, strain hardening and the formation of microcracks. Although the residual strain and strain hardening are a result of the nature of the polymer phase, the formation of microcracks is only observed upon the addition of the particulate phase.

It is demonstrated that healing of this early stage fatigue damage occurs in two different steps. Firstly, the deformation is restored by the free-shrinkage of the polymer. At temperatures below the melt temperature, the polymer network is healed and the fatigue induced strain hardening is reset. Secondly, only at temperatures above the melting point of the polymer phase, microcracks are sealed. It is shown that the thermally induced free-shrinkage in these polymers does not depend on the presence of ionic clusters, but that the ability to heal cracks in composite structures is reserved for ionomers that contain a sufficient amount of ionic clusters which guarantees an acceptable level of mechanical stability during healing. This implies that ionomers need to be thermally treated at above-the-melt temperatures in order to heal all the early stage damage that is induced upon fatigue loading.

## Figures and Tables

**Table 1:** Overview of the plateau moduli at different temperatures and the supramolecular bond lifetime at 110 °C for all polymer matrix blends.

Parameters	Zn-EMAA	EMAA	Zn-EMAA/EMAA	Zn-EMAA/AA
*G*_n_ at 80 °C (Pa)	8.2 × 10^6^	3.9 × 10^4^	1.2 × 10^6^	7.2 × 10^6^
*G*_n_ at 110 °C (Pa)	>6.4 × 10^5^	>2.8 × 10^3^	>4.4 × 10^5^	>3.8 × 10^5^
τ_b_ at 110 °C (s)	31	0.13	6.3	2.0

## Supplementary Materials

The following are available online at www.mdpi.com/2073-4360/8/12/436/s1. S1. Model Description; and S2. Effect of Fe_3_O_4_ Particles on Matrix Properties.

## References

[1] Van der Zwaag S. (2007). An introduction to material design principles: Damage prevention versus damage management. Self-Healing Materials an Alternative Approach to 20 Centuries of Materials Science.

[2] Van der Zwaag S., Grande A.M., Post W., Garcia S.J., Bor T.C. (2014). Review of current strategies to induce self-healing behaviour in fibre reinforced polymer based composites. Mater. Sci. Technol..

[3] Toohey K.S., Hansen C.J., Lewis J.A., White S.R., Sottos N.R. (2009). Delivery of two-part self-healing chemistry via microvascular networks. Adv. Funct. Mater..

[4] White S.R., Moore J.S., Sottos N.R., Krull B.P., Santa Cruz W.A., Gergely R.C.R. (2014). Restoration of large damage volumes in polymers. Science.

[5] Billiet S., Hillewaere X.K.D., Teixeira R.F.A., Du Prez F.E. (2013). Chemistry of crosslinking processes for self-healing polymers. Macromol. Rapid Commun..

[6] Garcia S.J. (2014). Effect of polymer architecture on the intrinsic self-healing character of polymers. Eur. Polym. J..

[7] Zhong N., Post W. (2015). Self-repair of structural and functional composites with intrinsically self-healing polymer matrices: A review. Compos. A Appl. Sci. Manuf..

[8] Hohlbein N., Shaaban A., Schmidt A.M. (2015). Remote-controlled activation of self-healing behavior in magneto-responsive ionomeric composites. Polymer.

[9] James N.K., Lafont U., van der Zwaag S., Groen W.A. (2014). Piezoelectric and mechanical properties of fatigue resistant, self healing pzt-ionomer composites. Smart Mater. Struct..

[10] Sundaresan V.B., Morgan A., Castellucci M. (2013). Self-healing of ionomeric polymers with carbon fibers from medium-velocity impact and resistive heating. Smart Mater. Res..

[11] Grande A.M., Castelnovo L., Landro L.D., Giacomuzzo C., Francesconi A., Rahman M.A. (2013). Rate-dependent self-healing behavior of an ethylene-co-methacrylic acid ionomer under high-energy impact conditions. J. Appl. Polym. Sci..

[12] Grande A.M., Coppi S., Di Landro L., Sala G., Giacomuzzo C., Francesconi A., Rahman M.A. An experimental study of the self-healing behavior of ionomeric systems under ballistic impact tests. Proceedings of the SPIE Optics and Photonics.

[13] Kalista S.J., Ward T.C., Oyetunji Z. (2007). Self-healing of poly(ethylene-co-methacrylic acid) copolymers following projectile puncture. Mech. Adv. Mater. Struct..

[14] Varley R.J., van der Zwaag S. (2008). Towards an understanding of thermally activated self-healing of an ionomer system during ballistic penetration. Acta Mater..

[15] Varley R.J., van der Zwaag S. (2010). Autonomous damage initiated healing in a thermo-responsive ionomer. Polym. Int..

[16] Luo X., Mather P.T. (2013). Shape memory assisted self-healing coating. ACS Macro Lett..

[17] Rodriguez E.D., Luo X., Mather P.T. (2011). Linear/network poly(ε-caprolactone) blends exhibiting shape memory assisted self-healing (smash). ACS Appl. Mater. Interfaces.

[18] Vega J.M., Grande A.M., van der Zwaag S., Garcia S.J. (2014). On the role of free carboxylic groups and cluster conformation on the surface scratch healing behaviour of ionomers. Eur. Polym. J..

[19] Wang C.H., Sidhu K., Yang T., Zhang J., Shanks R. (2012). Interlayer self-healing and toughening of carbon fibre/epoxy composites using copolymer films. Compos. A Appl. Sci. Manuf..

[20] Dolog R., Weiss R.A. (2013). Shape memory behavior of a polyethylene-based carboxylate ionomer. Macromolecules.

[21] Scogna R.C., Register R.A. (2009). Plastic deformation of ethylene/methacrylic acid copolymers and ionomers. J. Polym. Sci. B Polym. Phys..

[22] Slutsker A.I., Vettegren V.I., Kulik V.B., Hilarov V.L., Polikarpov Y.I., Karov D.D. (2015). Detailing of deformation processes in polymeric crystals. Phys. Solid State.

[23] Lacks D.J., Rutledge G.C. (1994). Mechanisms for axial thermal contraction in polymer crystals: Polyethylene vs. isotactic polypropylene. Chem. Eng. Sci..

[24] Kalista S.J., Pflug J.R., Varley R.J. (2013). Effect of ionic content on ballistic self-healing in emaa copolymers and ionomers. Polym. Chem..

[25] Amamoto Y., Otsuka H., Takahara A., Matyjaszewski K. (2012). Self-healing of covalently cross-linked polymers by reshuffling thiuram disulfide moieties in air under visible light. Adv. Mater..

[26] Canadell J., Goossens H., Klumperman B. (2011). Self-healing materials based on disulfide links. Macromolecules.

[27] Lafont U., Van Zeijl H., van der Zwaag S. (2012). Influence of cross-linkers on the cohesive and adhesive self-healing ability of polysulfide-based thermosets. ACS Appl. Mater. Interfaces.

[28] Adzima B.J., Kloxin C.J., Bowman C.N. (2010). Externally triggered healing of a thermoreversible covalent network via self-limited hysteresis heating. Adv. Mater..

[29] Corten C.C., Urban M.W., Shelby F. (2009). Repairing polymers using an oscillating magnetic field. Adv. Mater..

[30] Duenas T., Enke A., Chai K., Castellucci M., Sundaresan V.B., Wudl F., Murphy E.B., Mal A., Alexandar J.R., Corder A. (2010). Smart self-healing material systems using inductive and resistive heating. ACS Symp. Ser..

[31] Brown E.N., White S.R., Sottos N.R. (2005). Retardation and repair of fatigue cracks in a microcapsule toughened epoxy composite—Part ii: In situ self-healing. Compos. Sci. Technol..

[32] Jones A.S., Rule J.D., Moore J.S., Sottos N.R., White S.R. (2007). Life extension of self-healing polymers with rapidly growing fatigue cracks. J. R. Soc. Interface.

[33] Neuser S., Michaud V. (2014). Fatigue response of solvent-based self-healing smart materials. Exp. Mech..

[34] Yuan Y.C., Rong M.Z., Zhang M.Q., Yang G.C., Zhao J.Q. (2011). Self-healing of fatigue crack in epoxy materials with epoxy/mercaptan system. Express Polym. Lett..

[35] Luterbacher R., Trask R.S., Bond I.P. (2015). Static and fatigue tensile properties of cross-ply laminates containing vascules for self-healing applications. Smart Mater. Struct..

[36] Varley R.J., Shen S., van der Zwaag S. (2010). The effect of cluster plasticisation on the self healing behaviour of ionomers. Polymer.

[37] Hohlbein N., von Tapavicza M., Nellesen A., Schmidt A.M. (2013). Self-healing ionomers. Self-Healing Polymers.

[38] Tadano K., Hirasawa E., Yamamoto H., Yano S. (1989). Order-disorder transition of ionic clusters in ionomers. Macromolecules.

[39] Eisenberg A., Hird B., Moore R.B. (1990). A new multiplet-cluster model for the morphology of random ionomers. Macromolecules.

[40] Loo Y.L., Wakabayashi K., Huang Y.E., Register R.A., Hsiao B.S. (2005). Thin crystal melting produces the low-temperature endotherm in ethylene/methacrylic acid ionomers. Polymer.

[41] Bose R.K., Hohlbein N., Garcia S.J., Schmidt A.M., van der Zwaag S. (2015). Connecting supramolecular bond lifetime and network mobility for scratch healing in poly(butyl acrylate) ionomers containing sodium, zinc and cobalt. Phys. Chem. Chem. Phys..

[42] Fayolle B., Richaud E., Colin X., Verdu J. (2008). Review: Degradation-induced embrittlement in semi-crystalline polymers having their amorphous phase in rubbery state. J. Mater. Sci..

