# Supplementary material for: Healing of Early Stage Fatigue Damage in Ionomer/Fe3O4 Nanoparticle Composites
Source: Polymers (Basel). 2016 Dec 15;8(12):436. doi: 10.3390/polym8120436 (PMC6432088; doi:10.3390/polym8120436)
Supplement: Supplementary file 1 [file polymers-08-00436-s001.pdf]

Supplementary Materials: Healing of Early Stage Fatigue Damage in Ionomer/Fe_3O_4 Nanoparticle Composites

Wouter Post, Ranjita K. Bose, Santiago J. García and Sybrand van der Zwaag

S1. Model Description

The temperature of the tested specimen upon inductive heating was monitored with a FLIR A655sc infrared camera. Since this method only detects the surface temperature of the ionomer composites, a COMSOL Multiphysics 5.2 model was used to derive a relation between the measured surface temperature and the desired healing temperature within the bulk of the polymer sample. The used model is a stationary heat transfer model that correlates the measured surface temperature to the bulk healing temperature based on the thermal conductivity of the materials used.

The base of the model is a unit cell that consists of a cube containing a sphere in its centre that occupies 10 vol % of the cubic shape. This unit cell was then used to construct a 3 × 3 × 3 matrix resulting in a cubic structure with 27 equally divided spheres in its body. This cubic structure was placed in another cubic shape with a volume that is 159% larger than the matrix. In the model, the matrix represents the polymer composite and the spheres act as the iron oxide fillers. The surrounding cube represents the air that cools the specimen during heating. The "heat transfer in solids" physics module, available in COMSOL Multiphysics 5.2, was then applied to the developed geometry. The input data used for the polymer and particle phase are depicted in Table S1:

Table S1. Input parameters for heat transfer model used to correlate measured surface temperature to the overall bulk temperature.

Parameters	Surlyn 9520	Iron Oxide
Thermal conductivity (W/(m·K)	0.246	50
Density (kg/m^3)	950	5.1
Heat Capacity at constant pressure J/(kg·K)	2100	450

The surrounding layer of air was given the pre-programmed input parameters that are available in COMSOL Multiphysics 5.2. It is experimentally observed that, upon the application of a specific inductive field, the temperature of the particulate composite reaches a steady state within 5 min. Therefore a temperature profile is created by setting temperature boundary conditions on both the filler material and the surrounding air. The temperature of air was set at 293.15 K whereas the temperature of the spheres was varied from 343.15 to 473.15 K. Based on these input parameters the temperature gradient along the edge of the matrix structure is calculated as it represents the composite surface that is measured with the IR camera. The edge values (corner points of the matrix) of these temperature gradients are then considered to represent the surface temperature and are plotted versus the set boundary temperature of the particles, which is found to correspond to the uniform bulk temperature of the material. The resulting plot is shown in Figure S1.

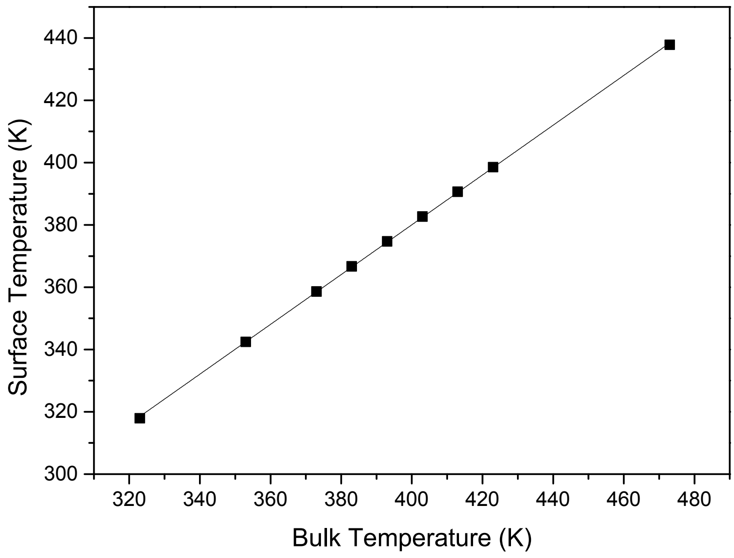

Figure S1. Calculated surface temperature versus bulk temperature.

From the calculated data the following linear relation was derived:

$$Surface\ Temperature\ = 0.80 * Bulk\ Temperature + 60.33 \quad \text{(S1)}$$

The empirical Equation (1) relates the measured surface temperature (in K) to the bulk temperature of the polymer and is used to determine the experimental healing temperatures.

S2. Effect of Fe_3O_4 Particles on Matrix Properties

The effect of the Fe_3O_4 particles on the ionomer matrix was investigated by tensile testing using an Instron Model 3365 universal testing systems equipped with a 1 kN load cell. Dog-bone micro-tensile specimens were stretched at 1 mm/s at room temperature. The tensile curves of a Zn-EMAA specimen with and without particle loading are shown in Figure S2.

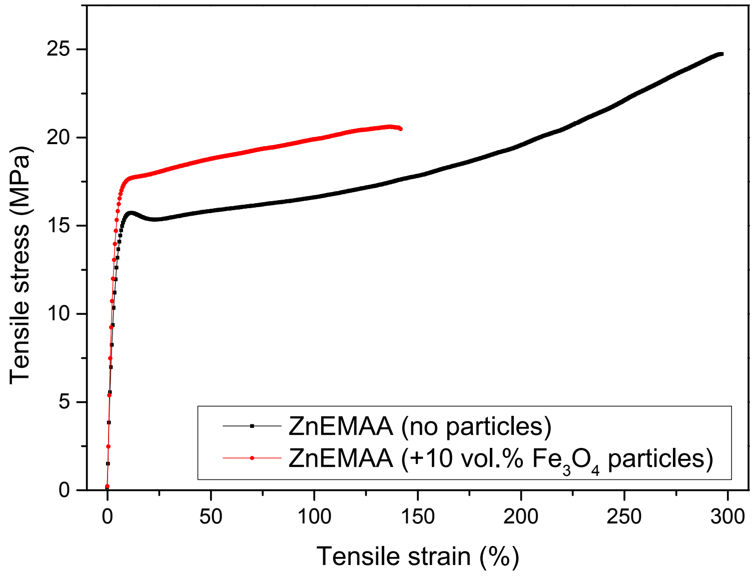

Figure S2. Stress-strain curves of the Zn-EMAA ionomer with and without the addition of particles.

Based on the tensile response in Figure S2 the yield strength, ultimate strength, Young's modulus and strain at break were determined. These values are determined for all four polymer blends and their values are depicted in Table S2.

Table S2. Overview of mechanical properties of the ionomer nanoparticle composites and their non-particle counterparts.

Polymer	Filler	Yield strength (MPa)	Ultimate strength (MPa)	Young's modulus (MPa)	Strain at break (%)
Zn-EMAA	No filler	15.7	24.7	365	297
	10 vol % Fe_3O_4	17.6	20.6	431	142
EMAA	No filler	8.7	23.5	108	455
	10 vol % Fe_3O_4	11.1	18.4	250	195
Zn-EMAA/EMAA	No filler	12.4	22.5	252	331
	10 vol % Fe_3O_4	13.6	19.5	324	179
Zn-EMAA/AA	No filler	10.3	14.2	222	267
	10 vol % Fe_3O_4	14.0	15.7	327	45
